# Intermittent Fasting: The Choice for a Healthier Lifestyle

**DOI:** 10.7759/cureus.2947

**Published:** 2018-07-09

**Authors:** Kavitha Ganesan, Yacob Habboush, Senan Sultan

**Affiliations:** 1 Internal Medicine, Orange Park Medical Center, Jacksonville, USA; 2 Endocrinology, Orange Park Medical Center, Jacksonville, USA

**Keywords:** intermittent fasting, weight loss, exercise

## Abstract

Obesity is a worldwide epidemic due to the availability of many unhealthy food options and limited physical exercise. Restriction of the daily food intake results in weight loss, which is also associated with better health outcomes including triglycerides, total cholesterol, low-density lipoprotein cholesterol, blood pressure, glucose, insulin, and C-reactive protein. Our aim is to briefly discuss the effects of intermittent fasting on weight and other biochemical markers mentioned previously. The study is designed as a systematic review according to the Preferred Reporting Items for Systematic Reviews and Meta-Analyses (PRISMA) checklist. To assess the effectiveness of intermittent fasting, related studies were reviewed between 2000 and 2018 and 815 studies were identified. Only four articles met the preset inclusion and exclusion criteria. All four studies have shown a significant decrease in fat mass with P-values <0.01. It was also noted that some biochemical markers were significantly reduced such as the reduction in low-density lipoprotein and triglyceride with P-values < 0.05. Other biochemical markers had inconsistent results. Based on the qualitative analysis, intermittent fasting was found to be efficient in reducing weight, irrespective of the body mass index. Further studies are needed to assess the ability to maintain the lost weight without regaining it and the long-term effects of such dietary changes.

## Introduction and background

In recent years, people worldwide have developed an increased popularity for weight loss program, diet plans and weight maintenance programs with little research done on the effectiveness of those programs. Meanwhile, obesity has been increasing in prevalence due to many social determinants such as easy access to various fast foods, and lack of physical activity [1]. In 2016, World Health Organization (WHO) reported that more than 1.9 billion people in the world were overweight and over 650 million people were obese which has tripled in number since 1975 [1]. Also, obesity is a known risk factor for many metabolic disorders like coronary heart disease, malignancies, osteoarthritis and respiratory disorders [2].

As per the meta-analysis done by Galani and Schneider, it was suggested that lifestyle changes are one of the most effective methods in reducing weight and the risks for cardiovascular diseases [3]. Weight loss usually leads to the improvement in the overall wellbeing of patients and their biomarkers such as systolic and diastolic blood pressure, blood sugar, insulin, total serum cholesterol, inflammatory biomarkers and low-density lipoprotein (LDL) [3]. There are many forms of diet and exercises programs available for weight loss, however, one of the least recognized diet changes is the alternate day fasting (ADF) which includes eating 20% of energy requirements on a fast day and then consume food ad libitum on the feed days which has been suggestive to be highly effective for weight loss.

The aim of our systematic review is to summarize the effects of ADF/intermittent fasting on weight loss, the influence on biomarkers, and cardiovascular risk factors thereby assessing it as a choice for a healthier lifestyle.

## Review

Methods

Study Design

The design of the study is a rapid qualitative systematic review in line with the Preferred Reporting Items for Systematic Reviews and Meta-Analyses (PRISMA) checklist. A literature review was performed independently by two reviewers, Kavitha Ganesan (KG) and Yacob Habboush (YH). The authors choose a wide range of search engines to use including PubMed, Medline, Ovid, EBSCO, Clinical Key and Google Scholar. Set words and terms were used to search for publications, including ‘intermittent fasting’, ‘weight loss’, and ‘low calorie’, to which we applied relevant subheading such as ‘fasting’, ‘diet’, ‘intermittent’, ‘overweight’ and ‘obese’. The search was limited by using the following filters: Human subjects, English language, and since the year 2000. Irrelevant publications were eliminated from the title of those studies which did not include the word fasting or dietary restriction. The present study is based on a qualitative approach to assessing the effectiveness of intermittent fasting in reducing body weight and adopting a healthier lifestyle. Therefore, the study design did not include the calculation of the quantitative measures.

Study Selection

The inclusion criteria for publications included any published article assessing intermittent fasting in 18-year-old patients or older, randomized controlled trials, studies measuring the effect of intermittent fasting on body weight as a primary outcome, and body mass index. We also assigned the Grading of Recommendations Assessment, Development and Evaluation (GRADE) score of four or higher as an evaluation measure of the publications overall quality [4]. Exclusion criteria included any study with participants younger than 18 years of age, any study that is not a randomized clinical, and those studies that did not score four or higher on the GRADE framework. Table 1 shows the four selected studies with definitions of the interventions.

**Table 1:** Types of interventions.

Author/year	Intervention type	Detention of intervention type
Varady K. et al. 2013 [5]	Alternate day fasting	A “fast day” where individuals consume 25% of energy needs, alternated with a “feed day” where subjects eat ad libitum.
Bhutani S. et al.2013 [6]	Alternate day fasting	A “fast day” where individuals consume 25% of energy needs, alternated with a “feed day” where subjects eat ad libitum.
	Exercise	Moderate intensity exercise program three times per week under supervised conditions using an age-predicted heart rate maximum with a heart rate monitor.
	Combined	Combination of alternate day fasting and exercise.
Heilbronn L. et al. 2005 [7]	Alternate day fasting	A “fast day” where individuals consume 25% of energy needs, alternated with a “feed day” where subjects eat ad libitum followed by two consecutive days a “feast” day and following a “fast” day.
Lantz H. et al. 2003 [8]	Very low-calorie diets	Daily energy intake of 450 kcal followed by refeeding phase, in which ordinary food was gradually introduced. For the remaining treatment period all the patients were recommended an individualized hypocaloric diet of 500 kcal day.

Outcomes

The outcome of interest is the effectiveness of intermittent fasting on weight loss and other biomarkers, while also assessing for the overall quality of the selected clinical trials to ensure high qualitative systematic review.

Results

Search Results and Study Characteristics

**Figure 1:**
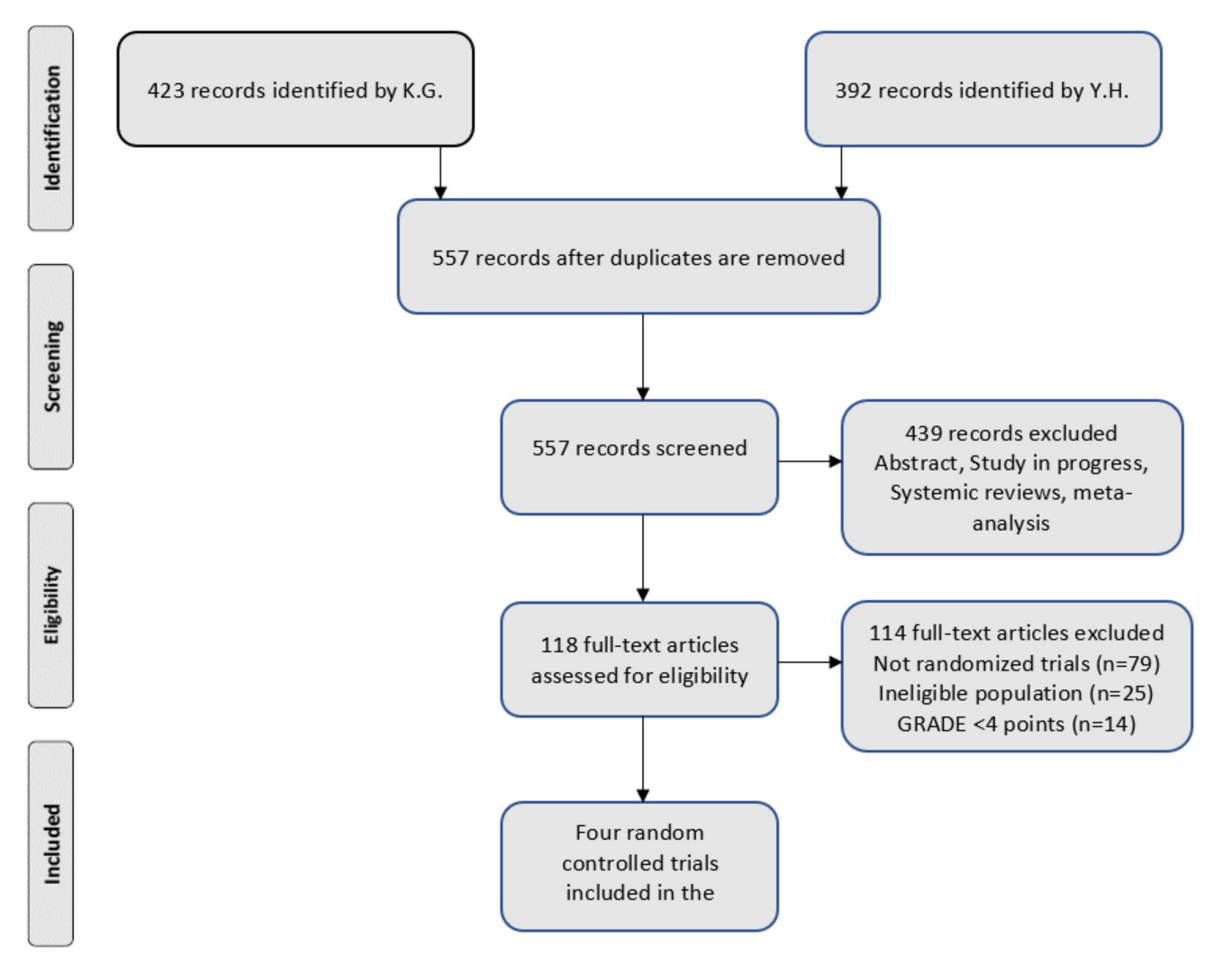
PRISMA flow diagram. PRISMA: Preferred Reporting Items for Systematic Reviews and Meta-Analyses; K.G.: Kavitha Ganesan; Y.H.: Yacob Habboush; GRADE: Grading of Recommendations Assessment, Development and Evaluation.

Both authors, KG and YH, cumulatively identified 815 studies since 2000 with a Kappa score of 0.689, which signifies a good strength of agreement. Of those, 258 articles were removed since they were duplicates. Further, 439 studies were excluded (abstract, study in progress, systematic reviews, and meta-analysis). Then we assessed 118 articles for eligibility. Figure 1 shows the PRISMA flow diagram. Finally, we included four randomized controlled trials in our qualitative systematic review. Table 2 provides the demographic information about the selected studies.

**Table 2:** Study characteristics of reviewed random controlled trials. BMI: Body mass index; RCT: Randomized controlled trial; US: United States; kg: kilogram; kg/m^2^: kilogram per meter square.

Author/year	Study design	Location	Number of participants in each group	Mean age per group	Diet only/Exercise only/Combination	Average weight per group (kg)	Average BMI per group (kg/m^2^)	Intervention period (weeks)
Varady K. et al. 2013 [5]	RCT	US	15, 15	47, 48	Diet only	77, 77	26, 26	12
Bhutani S. et al.2013 [6]	RCT	US	18, 25, 24, 16	45, 42, 42, 49	Diet only/Exercise only/Combination	91, 94, 93, 93	35, 35, 35, 35	12
Heilbronn L. et al. 2005 [7]	RCT	US	8, 8	34, 30	Diet only	80.6, 59.7	25.2, 22.6	3
Lantz H. et al. 2003 [8]	RCT	Sweden	161, 173	41.9, 41.4	Diet only	114.2, 114.4	39.9, 40.1	104

Outcomes

A randomized controlled trial by Varady et al. [5] evaluated the role of ADF to lose weight in overweight and normal weight individuals. Subjects had a feed day alternated with a fast day with 25% of total energy intake. Alternate day fasting group has a decrease in fat mass (P < 0.001) by 3.6 ± 0.7 kg, C-reactive protein (13 ± 17%, P < 0.05), leptin and triacylglycerol (TG) concentrations (20 ± 8%, P < 0.05), increase in low density lipoprotein (LDL) particle size (4 ± 1, P < 0.01) and plasma adiponectin (6 ± 10%, P < 0.01) while leptin decreased (40 ± 7%, P < 0.05) when compared to the control group [5]. However, there was no change in low-density lipoprotein, high-density lipoprotein, and homocysteine at the end of study period. Cases and controls did not have any issues in adhering to the study diet for 12 weeks. Reported hyperphagia on the feed day in turn leads to overall higher energy restriction throughout the study period which was a major cause of the weight loss. Dietary satisfaction and sense of feeling fullness increased at the end of 12 weeks which may have played a role in adherence to diet in the long-term. Few limitations of this study were the low number of study subjects (15 patients in each group), no physical activity measurement, the possibility of under-reporting of dietary intake as the reports were taken via food records [5].

The second study done by Bhutani et al. [6] measured the combination of ADF and exercise which was found to be superior to fasting or exercise alone in terms of lipid levels and changes in body mass composition. Sixty-four obese patients were divided into four groups as mentioned in Table 2. There was a reduction in body weight in the exercise only group and the combination groups. Moreover, the combination group had a reduction in LDL (5%, P < 0.05), the proportion of small high-density lipoprotein (HDL) particles (P < 0.01), fat mass, and waist circumference, however, the lean mass remained the same. There was also an increase in LDL particle size in ADF only group and the combination group. The dietary intervention had two periods; a controlled feeding period (25% of baseline energy requirements) for four weeks, and a self-selected feeding period (food ad libitum) for next eight weeks [6]. Participants in the exercise group and combination group had a moderate intensity supervised exercise program three times per week for 12 weeks. Initially, they were exercising for 25 minutes which was gradually increased to the duration of 40 minutes by week 10. Few limitations of the study were that it might take more than 16 weeks for HDL cholesterol to be altered supervised endurance training. Also, exercise frequency and intensity mentioned in the study may not be sufficient to change the risk indicators of coronary artery disease [6].

Heilbronn et al. [7] conducted a study to determine whether ADF is a good model of dietary restriction in non-obese people and whether there is any improvement in longevity of the people. They recruited 16 people with eight men and eight women who fasted every other day for 22 days. Individuals lost about 2.5% (P 0.001) of their initial body weight and 4% of initial fat mass (P 0.001). Fat oxidation was increased more than or equal to 15 grams in non-obese subjects, but the hunger did not decrease on non-fasting days. They recommended that adding one small meal on fasting days will make alternate fasting more feasible. Resting metabolic rate and respiratory quotient did not change significantly from baseline to day 21 and 22 of ADF. There was no significant change in ghrelin and glucose from baseline in alternate fasting subjects while fasting insulin was decreased by 57% (P < 0.001). Level of physical activity varied among the subjects. Seven people were sedentary, three had moderate activity level and three people had very active life (four to five times of exercise per week) [7].

The last study is a two-year randomized controlled trial by Lantz et al. [8] consisted of 334 patients who were evaluated for the effect of intermittent versus on-demand intake of very low-calorie diet (VLCD) for maintenance of weight in obese patients for 16 weeks. The intermittent group had VLCD for two weeks every third month, while on-demand group patients had VLCD when the body weight has passed the individual cut off level. They had hypocaloric diet during other periods. VLCD-based regimen had clinically significant weight loss after two years (7.0 ± 11.0 kg (6.2 ± 9.5%) with P < 0.001). Many risk factors for cardiovascular disease were improved during the first year including HDL, LDL, and insulin were improved significantly at the end of two years of the study. Calorie level of VLCD was 450 kilocalories. In the refeeding phase of three weeks, ordinary food was introduced gradually, then subjects were given individualized hypocaloric diet (500 calories per day) for up to two years [8]. Tables 3 and 4 summarize some of the significant outcomes.

**Table 3:** The mean change in weight. ADF: Alternate day fasting; FM: Fat mass; FFM: Fat free mass; SEM: Standard error of mean; CI: Confidence interval.

Study	Groups	FM/FFM	Mean mass change from baseline/Kg (SEM or CI)	P-value
Varady K. et al. [5]	ADF vs. Control	FM	-3.6 (± 0.7)	<0.001
		FFM	0	-
Bhutani S. et al. [6]	ADF	FM	-2 (± 1)	0.008
		FFM	-1 (± 1)	0.031
	Exercise	FM	-1 (± 0)	0.182
		FFM	-1 (± 0)	0.321
	Combination	FM	-5 (± 1)	<0.001
		FFM	0 (± 1)	0.299
	Control	FM	0 (± 1)	0.570
		FFM	-1 (± 1)	0.693
Heilbronn L. et al. [7]	ADF vs. Control	FM	-0.8	<0.001
		FFM	-0.6	<0.05
Lantz H. et al. [8]	ADF vs. Control	FM	-6.46 (-8.1; -4.8)	<0.05
		FFM	-2.64 (-5.5; -3.6)	<0.05

**Table 4:** Changes in lipid and blood pressure compositions. LDL: Low-density lipoprotein; HDL: High density lipoprotein; TG: Triglyceride; SBP: Systolic blood pressure; DBP: Diastolic blood pressure; ADF: Alternate day fasting.

Study	Groups	Total cholesterol (mg/dl)	LDL (mg/dl)	HDL (mg/dl)	TG (mg/dl)	SBP (mm Hg)	DBP (mm Hg)
Varady K. et al. [5]	ADF	-26 ± 6	-18 ± 6	-2 ± 3	-22 ± 11	-25 ± 5	4 ± 1
	Control	-9 ± 5	-9 ± 4	1 ± 2	10 ± 7	-9 ± 5	-2 ± 1
Bhutani S. et al. [6]	ADF	7 ± 4	-1 ± 6	0 ± 4	6 ± 6	-3 ± 1	-2 ± 2
	Exercise	0 ± 3	0 ± 5	2 ± 3	7 ± 6	2 ± 2	0 ± 2
	Combination	-2 ± 5	-12 ± 5	18 ± 9	13 ± 11	-2 ± 2	0 ± 3
	Control	1 ± 4	3 ± 5	8 ± 5	5 ± 7	-2 ± 3	-2 ± 3
Heilbronn L. et al. [7]	ADF vs. Control	-	-	-	-	-	-
Lantz H. et al. [8]	ADF vs. Control	-0.1	-0.2	0.2	-0.1	0 ± 3	0 ± 2

Discussion

A systematic review of the four studies discussed showed that intermittent fasting was effective for short-term weight loss. However, there was increased variability in our included studies, ranging from 16 to 334 participants with a follow-up period ranging from three weeks to 104 weeks. Baseline characteristics of the study population were also different in terms of body mass index which included normal weight subjects, overweight and obese subjects. Mode of interventions was also different for each individual study as shown in Table 1 [5-8].

A systematic review by Davis et al. found that dietary plans had significant weight loss in intermittent fasting groups [9]. Seimon et al. in his systematic review found that intermittent fasting diet was as effective as daily restriction of calories both for short and long-term interventions [10]. Most common issues with continuous calorie restriction diet are that restriction of food continuously is a trigger for higher hunger and additional eating. In these situations, ADF is a better solution which might be an optimal solution so that people can eat in their usual ways on non-fast days. This also depends on the type of the ADF used which minimizes the fatigue associated with continuous calorie restriction [11].

There are limited data available in the literature regarding the consistency, tolerability, and safety of ADF among the general population when used as an intervention for weight loss. Varady et al. mentioned that minimal adverse effects such as mild headaches and constipation were experienced in three patients and only one patient was dropped out of the study because of difficulty in adhering to the diet [5]. A systematic review by Heilbronn and Ravussin [12] mentioned that there were few adverse effects related to the study such as lightheadedness and constipation. Few people were irritable during fast days. Lantz et al. study had a dropout rate of about 65%, also there was a lack of very low-calorie diet free treatment group during the maintenance phase to serve as a control group [8].

The dietary program called continuous energy restriction (CER) is another effective mean of weight reduction in obese and normal weight individuals. It includes restriction of daily calorie intake by 15-60% of baseline energy requirements which is typically very hard to maintain over a long period of time because of the need to exercise self-regulation and tracking of the calories [13, 14]. In addition, CER may cause physiologic adaptations in the body to adapt for calorie restriction which might prevent further weight loss. Therefore, the alternate form of dieting intermittent energy restriction (IER) has been gaining popularity due to its superior efficacy [13, 14].

One form of IER is ADF which involves a fast-day where participants reduce or completely withhold any food intake and feed-day in which there is an ad libitum food consumption. The second form consists of a whole day fasting including complete fasting for one or two days in a week or with 25% of calorie intake in a day with no restriction of food for other days in a week. The third form is time restricted feeding where meals are eaten during specific times in the day, for example, from 8 am to 5 pm while people will remain on fasting for other hours in the same day [15].

Few biochemical changes associated with ADF in terms of weight loss change in mu (μ) opioid receptor characteristics, activation of the mesocorticolimbic dopamine system, and reduction of D2 dopamine receptor expression [16]. One of the proposed mechanisms for the small HDL particles to cause an increase in coronary heart disease may be related to alteration in the activity of lipases which are associated with the maturation and transformation of lipoproteins. Increase in proportion of the small LDL particles increases the risk of coronary artery disease by augmenting oxidization and increase in permeability of endothelial barrier [17, 18].

Advantages of ADF include subject satisfaction without food restriction, anxiety, and no reported hyperphagia. Subjects tend to watch what they eat even beyond the period of calorie restriction as they were used to ADF. People who either eat one or two meals per day or do not eat anything for long stretches of time may show better compliance of ADF diet which may result in greater weight loss [5-8].

Potential disadvantage of ADF is that it is not appropriate for individuals who are required to eat meals at regular intervals such as type 1 diabetes, pregnant and breastfeeding women, elderly, individuals with eating disorders and those in need of regular food intake to take medications. Weight loss generally plateau in six months, thus focus on weight maintenance after the initial period of weight loss is important by adherence to low-calorie diet and regular physical activity for a longer period of time [5-8].

There were few studies regarding alternating fasting that was conducted for a short duration of three weeks [19]. Redman et al. [20] conducted a study in obese people similar to Bhutani et al. [6] with a combined calorie restriction (reduction in energy intake by 25%) and supervised exercise program (moderate intensity exercise for about 60 minutes for five days in a week) for twelve weeks. It showed a weight loss of about 6% from the baseline in the study group. As per Bhutani et al. [6], combination therapy may cause retention of lean mass at the expense of fat mass during the energy restriction period which in turn helps to maintain resting metabolic rate thereby led to increases in energy burning capacity and weight loss. Seimon et al. mentioned that intermittent energy restriction is effective when compared to CER for short-term weight loss with three to five kilograms weight loss in approximately 10 weeks [10].

There are a few limitations associated with this systematic review. Studies that were published in other languages were excluded. Also, the location of these studies were university-based settings where participants had access to high-quality foods, dietician consultants, and counseling from behavioral health person. Thus, the external validity of these studies was doubtful in terms of applying it to the rural population who would like to achieve weight loss. It was difficult to ascertain for publication bias in our included primary studies, which may have reported successful interventions and selection bias as they might have enrolled subjects who had high motivation for adherence to diet. Lastly, our included studies were highly variable in terms of study design, the composition of the diet, amount of energy restriction, the timing of fasting period in a day and physical activity inclusion as mentioned in Table 2.

## Conclusions

The systematic review of the aforementioned four studies found that intermittent fasting was effective for short-term weight loss among normal weight, overweight and obese people. Randomized controlled trials with long-term follow-up period are needed to follow the adherence to diet and long-term maintenance of weight loss without regaining the lost weight. Future studies should also include specific subgroups of the population such as individuals with cardiovascular risk factors and type 2 diabetes mellitus as these patient population benefit more from weight loss which may modify the disease process. In summary, obesity and overweight is an international health crisis, and interventions such as ADF are needed to help people to achieve weight loss.
